# Dual cervical ossicles

**DOI:** 10.11604/pamj.2022.41.96.33502

**Published:** 2022-02-03

**Authors:** Sharfuddin Chowdhury

**Affiliations:** 1 Trauma Center, King Saud Medical City, Riyadh, Saudi Arabia

**Keywords:** Multiple trauma, spine, neck

## Image in medicine

A 29-year-old male, involved in a motorcycle accident 10-days prior and referred from a private hospital, presented with a history of abdominal pain, distension, and vomiting. On presentation, he was conscious, oriented, tachycardic with a heart rate of 112 beats/min, systolic blood pressure of 115 mmHg, tachypneic with a respiratory rate of 24/min, and febrile with a temperature of 38°C. After initial resuscitation, he underwent a Pan-computed tomography (CT) scan. Computed tomography abdomen revealed pneumoperitoneum, displaced fracture of left inferior pubic rami, and a moderate amount of free fluid with linear contrast extravasation along the dome of urinary bladder extended diffusely with intraperitoneal free fluids indicating the possibility of intraperitoneal bladder rupture. Cervical CT showed a rounded nondisplaced fracture of the C1 arch and a type 1 oblique odontoid minimally displaced fracture. Initial urea (BUN) of 22 mmol/L (reference range, 2.5 to 6.4 mmol/L) and serum creatinine of 412 umol/L (reference range, 62 to 115 umol/L) also supported intraperitoneal bladder injury. We performed an emergency laparotomy and repaired the bladder. His postoperative course was uneventful. He improved in general and a repeat CT cystogram on day 8 revealed no contrast leak. Concerning his cervical injury, a dilemma existed regarding fractures versus ossicles. The spinal surgeon requested a cervical magnetic resonance imaging (MRI), which showed no acute ligamentous injuries and a rare occurrence of dual cervical ossicles. In terms of his neck, he was asymptomatic. We removed his C- collar and discharged him from the hospital on day 11 post-admission.

**Figure 1:**
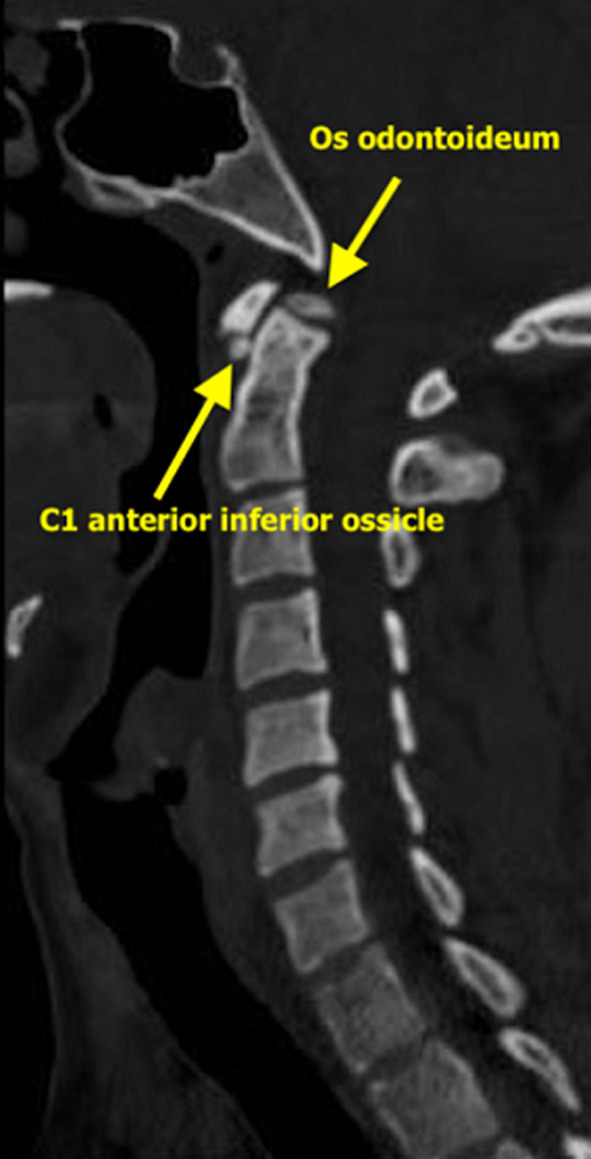
sagittal view of neck CT scan is showing dual cervical (C1-C2) ossicles

